# Draft genome assemblies of 12 *Enterobacter hormaechei* strains isolated from the North West province of South Africa

**DOI:** 10.1128/MRA.00757-23

**Published:** 2023-10-31

**Authors:** Lerato Lisbeth Njaki Makhetha, Khayalethu Ntushelo, Baitsholetsi Gloria Mokolopi, James Wabwire Oguttu, Christian Anayochukwu Mbajiorgu, Goitsemang Makete, Tshifhiwa Paris Mamphogoro

**Affiliations:** 1 Department of Agriculture and Animal Health, University of South Africa, Science Campus, Florida, South Africa; 2 Gastro-Intestinal Microbiology and Biotechnology Unit, Agricultural Research Council-Animal Production, Irene, Pretoria, South Africa; California State University, San Marcos, California, USA

**Keywords:** *Enterobacter hormaechei*, cattle, rumen microbial, antimicrobial resistance, tomato-associated bacteria, yield promoting bacterium

## Abstract

We present draft genome sequences of 12 *Enterobacter hormaechei* strains from feces isolated from cow rectums in the North West province, South Africa. The genome sizes ranged from 4.43 to 5.02Mb, with G + C contents of 55.5–56%, and contained 16–262 contigs. These data will contribute to our knowledge of the ecology and diversity of this species in South Africa.

## ANNOUNCEMENT

Cattle are an important economic commodity for most rural households in the North West province in South Africa. Moreover, cattle are also an important part of the ecosystem as grazers or consumers of plants especially grass. Therefore, the rumen of cattle harbor various microbes which may reflect the microbial diversity of an ecosystem (1). *Enterobacter hormaechei* is a plant growth and yield-promoting bacterium in tomato and okra plants, and it also enhances disease and salinity tolerance in tomatoes (2, 3).

The bacteria were isolated from cattle in Mogosane Village in the North West province, South Africa (25°45′30.6″S 25°33′43.9″E). Thirty-five cattle were selected for the study, and fecal samples were directly collected from the rectum of each cow using a sterile arm-length glove. The faeces samples were enclosed in sterile plastic containers and transported on ice to the laboratory. A loopful of each faeces sample was inoculated into Buffered Peptone Water medium; the mixture was serially diluted up to 10^−2^. The sample suspension was then inoculated onto McConkey agar and incubated for 18–24 h at 37°C under aerobic conditions. Distinct pink bacterial colonies formed on the plates were selected. Pure culture of the bacterial isolate was obtained by repeated streaking onto sterile Blood Agar (BA).

Genomic DNA was isolated from overnight cultures in BA using HighPure PCR template preparation kit (Roche Diagnostics, Mannheim, Germany; Cat No: 11796828001), following the manufacturer’s protocol. The extracted DNA concentration was measured using a NanoDrop (ThermoFisher Scientific, Carlsbad, CA, USA), while DNA quality was evaluated on 2% agarose gel. The paired-end (2 × 150 bp) libraries were generated using the Illumina TruSeq DNA Nano Preparation Kit (Illumina, San Diego, CA, USA); these libraries were sequenced on an Illumina Hiseq X instrument with standard operating procedures at Agricultural Research Council-Biotechnology Platform in South Africa, producing a total of 4,676,625–6,656,610 paired-end 2 × 150 bp reads. The sequences were analyzed via KBase platform (4). The quality of the reads was evaluated using FastQC v0.11.5 (5), while the raw Illumina paired-end reads were filtered to remove sequence adaptors and low-quality reads (quality score of <15) using Trimmomatic v0.36 (MINLEN:75) (6). The trimmed reads were then used for *de novo* assembly using SPAdes v3.13.0 (7). Contigs representing sequencing artifacts (<500 bp) were removed, and the post-filtered assemblies were used for the subsequent analysis. Assembly quality was assessed using QUAST v5.0.2 (8). While the genome completeness and contamination were evaluated using CheckM v1.0.18 (9).

Identification of each isolate was conducted using Kaiju v1.7.3 (10), and the results were visualized using Krona v2.7.1 (11). The contigs for each strain were annotated using NCBI’s Prokaryotic Genome Annotation Pipeline v6.3 (12). All software programs were run with default parameters. The genome sizes ranged from 4.43 to 5.02 Mb, and the G + C contents ranged from 55.5 to 56%. The data obtained provide support for future research regarding *E. hormaechei* epidemiology, phylogenetics, antimicrobial resistance, virulence, and plant growth-promoting gene content, which will be detailed in future publications.

**TABLE 1:** Metadata of the 12 sequenced *Enterobacter hormaechei* strains isolated from rectal fecal material of cattle from Mogosane Village in the North West province of South Africa

Strain name	Date of isolation	Isolation site (source)	Reference strain	GenBank accession no.	BioSample accession no.	SRA accession no.^*a*^	No. of reads	Assembly size (Mb)	G + C content (%)	No. of contigs	Coverage (X)	N50 contig (bp)	L50 contig	No. of genes (total)	No. of coding genes	No. of rRNAs	No. of tRNAs	No. of RNAs	No. of pseudo-genes
HCF1	14/06/2017	MVNWPSA^*b*^ (*Bos taurus* fecal sample)	*Enterobacter hormaechei* subsp. *xiangfangensis*	JAUHQK000000000	SAMN36291778	SRR25132773	4,676,625	4.51	55.5	25	312	302,776	5	4,281	4,156	7	66	81	44
HCF2	14/06/2017	MVNWPSA^*b*^ (*Bos taurus* fecal sample)	*Enterobacter hormaechei* subsp. *xiangfangensis*	JAUOLX000000000	SAMN36292261	SRR25132929	4,770,778	4.71	55.5	26	305	515,661	4	4,473	4,334	8	72	87	52
HCF3	14/06/2017	MVNWPSA^*b*^ (*Bos taurus* fecal sample)	*Enterobacter hormaechei* subsp. *xiangfangensis*	JAUOLV000000000	SAMN36292742	SRR25160155	5,340,394	4.56	55.5	30	353	456,289	4	4,394	4,267	7	76	89	38
HCF4	14/06/2017	MVNWPSA^*b*^ (*Bos taurus* fecal sample)	*Enterobacter hormaechei* subsp. *xiangfangensis*	JAUOLW000000000	SAMN36292822	SRR25133425	4,795,322	4.56	55.5	26	317	764,843	3	4,391	4,274	6	67	79	38
HCF5	14/06/2017	MVNWPSA^*b*^ (*Bos taurus* fecal sample)	*Enterobacter hormaechei* subsp. *xiangfangensis*	JAUIZV000000000	SAMN36315906	SRR25158114	6,238,972	4.70	55.5	28	400	441,216	5	4,470	4,335	8	68	83	52
HCF6	14/06/2017	MVNWPSA^*b*^ (*Bos taurus* fecal sample)	*Enterobacter hormaechei* subsp. *oharae*	JAUIZW000000000	SAMN36316627	SRR25159898	5,296,276	4.43	56	33	360	425,702	5	4,230	4,119	8	71	84	27
HCF7	14/06/2017	MVNWPSA^*b*^ (*Bos taurus* fecal sample)	*Enterobacter hormaechei* subsp. *xiangfangensis*	JAUIZX000000000	SAMN36317785	SRR25160397	5,966,971	4.59	55.5	27	392	484,492	4	4,370	4,245	8	72	86	39
HCF8	14/06/2017	MVNWPSA^*b*^ (*Bos taurus* fecal sample)	*Enterobacter hormaechei* subsp. *xiangfangensis*	JAUIZY000000000	SAMN36319008	SRR25181455	5,639,719	4.52	55.5	29	376	478,206	4	4,293	4,160	8	75	89	44
HCF9	14/06/2017	MVNWPSA^*b*^ (*Bos taurus* fecal sample)	*Enterobacter hormaechei* subsp. *steigerwaltii*	JAUKNV000000000	SAMN36346506	SRR25181904	4,876,499	5.02	55.5	262	292	81,556	18	4,929	4,800	8	77	91	38
HCF10	14/06/2017	MVNWPSA^*b*^ (*Bos taurus* fecal sample)	*Enterobacter hormaechei* subsp. *xiangfangensis*	JAUJUG000000000	SAMN36346815	SRR25210418	5,980,271	4.59	55.5	29	392	577,149	3	4,376	4,248	10	72	88	40
HCF11	14/06/2017	MVNWPSA^*b*^ (*Bos taurus* fecal sample)	*Enterobacter hormaechei* subsp. *steigerwaltii*	JAUJWX000000000	SAMN36378996	SRR25211045	5,820,958	4.55	56	16	385	640,068	3	4,311	4,200	7	72	84	27
HCF12	14/06/2017	MVNWPSA^*b*^ (*Bos taurus* fecal sample)	*Enterobacter hormaechei* subsp. *steigerwaltii*	JAUJWY000000000	SAMN36378998	SRR25211258	6,656,610	4.63	55.5	16	434	575,824	4	4,398	4,289	5	73	84	25

^*a*^ SRA**,** Sequence Read Archive.

^*b*^ MVNWPSA, Mogosane Village in the North West province in South Africa.

## Data Availability

The draft genome sequences of the 12 *Enterobacter hormaechei* isolates reported here are available at GenBank under the accession numbers listed in Table 1; these post-filtered assemblies were made publicly available on National Center for Biotechnology Information (NCBI). The NCBI BioProject accession number is PRJNA991313.
